# Genetic Signature of Resistance to White Band Disease in the Caribbean Staghorn Coral *Acropora cervicornis*

**DOI:** 10.1371/journal.pone.0146636

**Published:** 2016-01-19

**Authors:** Silvia Libro, Steven V. Vollmer

**Affiliations:** Marine Science Center, Northeastern University, 430 Nahant Road, Nahant, Massachusetts, United States of America; GEOMAR Helmholtz Centre for Ocean Research Kiel, GERMANY

## Abstract

Coral reefs are declining worldwide due to multiple factors including rising sea surface temperature, ocean acidification, and disease outbreaks. Over the last 30 years, White Band Disease (WBD) alone has killed up to 95% of the Caribbean`s dominant shallow-water corals—the staghorn coral *Acropora cervicornis* and the elkhorn coral *A*. *palmata*. Both corals are now listed on the US Endangered Species Act, and while their recovery has been slow, recent transmission surveys indicate that more than 5% of staghorn corals are disease resistant. Here we compared transcriptome-wide gene expression between resistant and susceptible staghorn corals exposed to WBD using *in situ* transmission assays. We identified constitutive gene expression differences underlying disease resistance that are independent from the immune response associated with disease exposure. Genes involved in RNA interference-mediated gene silencing, including Argonaute were up-regulated in resistant corals, whereas heat shock proteins (HSPs) were down-regulated. Up-regulation of Argonaute proteins indicates that post-transcriptional gene silencing plays a key, but previously unsuspected role in coral immunity and disease resistance. Constitutive expression of HSPs has been linked to thermal resilience in other *Acropora* corals, suggesting that the down-regulation of HSPs in disease resistant staghorn corals may confer a dual benefit of thermal resilience.

## Introduction

Coral reefs are the world’s most biodiverse marine ecosystems, hosting approximately 25% of all marine species [1]. Reef-building corals provide the foundation for these diverse communities, but are highly vulnerable to environmental stressors and anthropogenic impacts including rising sea surface temperatures, ocean acidification and disease outbreaks associated with climate change [2, 3]. It has been estimated that one third of world’s reef-building corals are at risk of extinction [4]. Recent studies indicate that some corals have a better ability to recover from bleaching events and disease outbreaks [5, 6] highlighting the importance of understanding the mechanisms of resilience and resistance in coral reef restoration and conservation strategies, but the mechanisms underlying coral resilience are not yet understood, especially the basis of disease resistance.

The Caribbean staghorn coral *A*. *cervicornis* declined dramatically due to White Band Disease (WBD) outbreaks starting in 1979 and is now considered at high extinction risk [7, 8]. Koch-Henle’s postulates for WBD have not yet been fulfilled, but multiple studies indicate that the pathogen is bacterial [9, 10] with *Vibrio charchariae* and *Rickettsia* CAR1 both shown to be associated with the disease [11–13]. The disease can be transmitted by direct contact, via the water if the coral is injured, and by animal vectors like the corallivorous snail *Coralliophila abbreviata* [14]. *In situ* transmission experiments indicate that more than 5% of staghorn corals are resistant to WBD infection [15]. RNA-seq analyses indicate that staghorn corals mount a vigorous immune response to WBD mediated by Pathogen Recognition Receptors (PRRs), apoptosis, production of Reactive Oxygen Species (ROS) and synthesis of eicoesanoids [16], but the genetic differences between disease resistant and susceptible corals are still unknown.

In order to identify genetic signatures underlying disease resistance in *Acropora cervicornis*, we used RNAseq to compare transcriptome profiles of resistant and susceptible staghorn corals genotypes that were exposed to WBD-infected (WBD grafts) tissue, healthy allogeneic tissue (healthy grafts) or left unexposed (no-graft controls). Transcriptome-wide comparisons of gene expression between resistant and susceptible corals revealed that none of the main immune pathways previously identified in WBD-infected staghorn corals [16] were associated to resistance. Instead, resistant corals up-regulated genes involved in RNA interference-mediated gene silencing, including Argonaute and NOD-like receptor family CARD domain containing 5 (NLRC5), and down-regulated heat shock proteins (HSPs) Interestingly, these differences between resistant and susceptible corals were independent from disease exposure indicating a constitutive basis for disease resistance in staghorn coral.

## Materials and Methods

Resistant and susceptible staghorn coral genotypes were identified using a series of *in situ* transmission experiments conducted in the region of Bocas Del Toro (Republic of Panama) during July 2005, Sept. 2005, May 2006, and Aug. 2006 as described in [15]. Staghorn coral colonies were tagged along permanent transects using numbered plastic tags. Each tagged coral colony was genotyped at five microsatellite loci [loci 166, 181, 182, 187, and 201; [15] after [17]] using modified PCR protocols [15] to identify unique staghorn genotypes. Resistance and susceptibility were assayed using *in situ* transmission experiments. Six replicate fragments were sampled from the parent colonies of each tagged coral genotype; five fragments were grafted to an active piece of WBD and the sixth fragment was grafted with a healthy piece of staghorn coral as a control. WBD transmission was monitored daily for the presence of WBD. The presence or absence of WBD was recorded for each fragment and used to calculate the probability of “resistance” (i.e. that no WBD transmission was observed) for each genotype after [15] using the binomial probability [i.e. Pr(No WBD Infection) = (1–average rate of transmission across genotypes)^#Replicate grafts per genotype^]. Genotypes that did not contract WBD in more than five replicate transmission attempts were statistically resistant, while susceptible genotypes were defined as genotypes that contracted WBD in more than 70% of the transmission attempts [15].

### Exposure experiment

**Fig 1:**
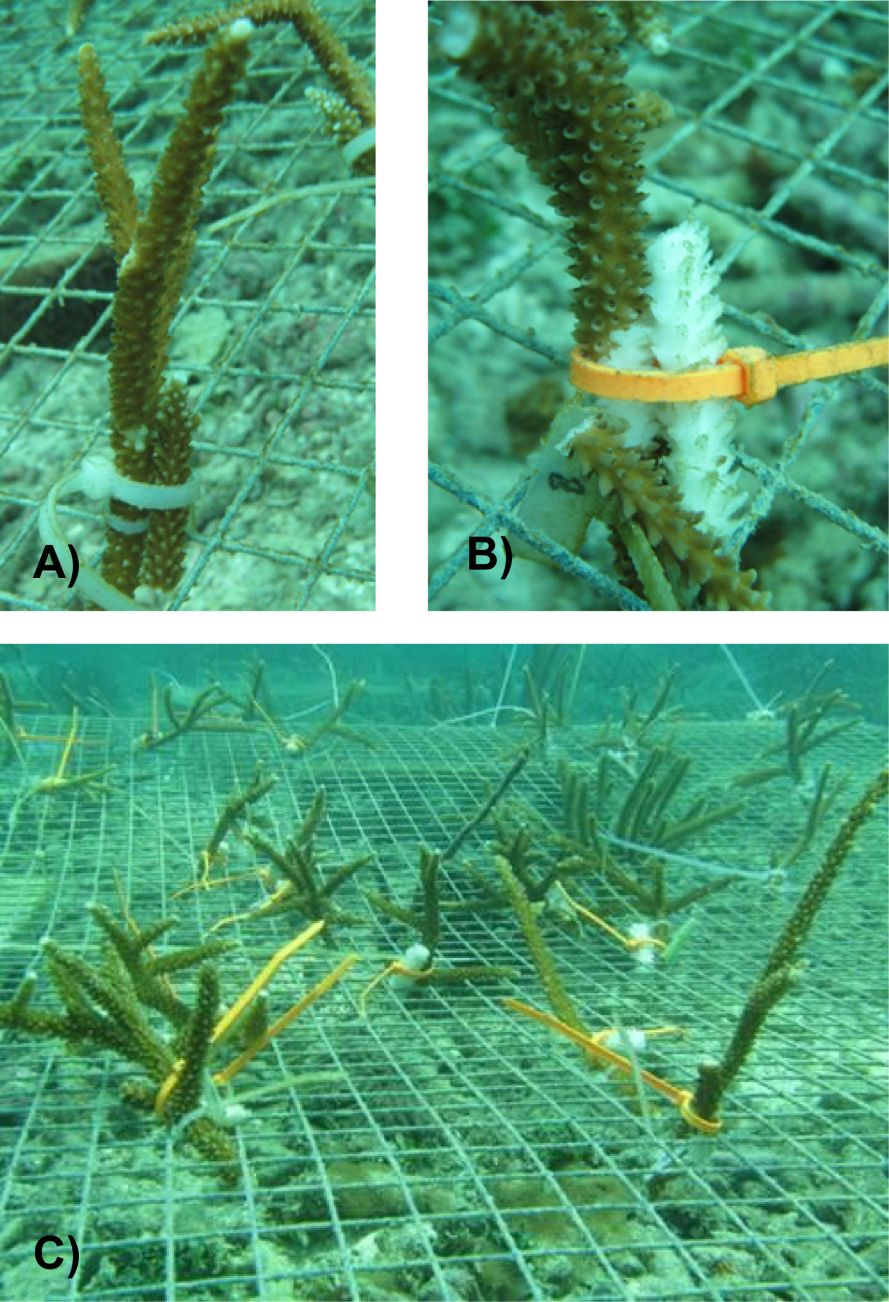
*In situ* transmission experiment. Replicate fragments of resistant and susceptible corals were exposed to allogeneic tissue and to active WBD using healthy grafts (a) and WBD grafts (b) and placed in common gardens (c).

The *in situ* exposure experiment used to profile the genetic response of resistant versus susceptible staghorn corals with RNAseq was conducted in common gardens located in Crawl Cay along the coasts of Isla Bastimentos (Bocas Del Toro, Republic of Panama) in July 2009. Four resistant and three susceptible genotypes of *A*. *cervicornis* identified in our previous *in situ* transmission assays were used in this experiment. For each genotype, five replicate fragments (15 cm tall) were collected from the tagged parent colonies in the field and placed in the 1.5 x 1.5 meter common garden for 24 hours to acclimate. One fragment from each genotype served as the no-graft control. Out of the remaining four fragments per genotype, three were grafted with active WBD infected tissue and two fragments were grafted with healthy (allogeneic) tissue (Fig 1).

Healthy and disease tissues used as grafts were sampled from distinct staghorn colonies in the adjacent reef. WBD transmission was monitored daily. One set of each experimental treatment (no graft control, healthy graft and disease graft) was collected for each genotype after three days of exposure and immediately flash frozen in liquid nitrogen and stored at -80°C for RNA analysis.

### RNA-seq library preparation

Total RNA was extracted using TriReagent (Molecular Research Center, Inc.) following the manufacturer's protocol. We obtained RNAseq data for four resistant and three susceptible genotypes. For the resistant genotypes, we obtain RNAseq data for three no graft controls, three healthy and four disease grafts in total. For the susceptible genotypes, we obtained RNAseq data for two no graft controls, two healthy and three disease grafts. The remaining fragments (one healthy and one disease graft from a resistant genotype, and one no graft control and one healthy graft from a susceptible genotype) were not included in this study due to issues with RNA quality.

RNAseq library preparation was performed with a modified Illumina mRNA-seq protocol as described in Libro et al [16]. Multiplexed paired-end libraries were sequenced on a Illumina GAII platform (Illumina, Inc, San Diego, California, USA) at the FAS Center for System Biology at Harvard University. De-multiplexing, read trimming and thinning were performed using custom Perl Scripts in the FASTX-Toolkit (http://hannonlab.cshl.edu/fastx_toolkit/). Reads were mapped to our published reference transcriptome [16] using RNAseq data obtained from 39 *A*. *cervicornis* and six *A*. *palmata* whole (holobiont) coral tissue samples [16]. The reference transcriptome was composed of 95,389 transcripts, of which 47,748 were identified as coral (e-value < 10^−10^) and 47,641 as non-coral (i.e. representing algae and symbiotic microorganisms, including zooxanthellae, fungi and protozoa) based on alignment against two *Acropora* zooxanthellae-free genomes [16, 18]. Functional annotation was performed using tBLASTx with an e-value cutoff less than 10^−5^ [19] against the Swiss-Prot and TReMBL protein databases [20]. Gene functions for the annotated transcripts were retrieved using Gene ontology (GO) terms and manually curated categories, to highlight presence of immune related domains or a putative immune role based on the literature. The reference transcriptome sequences are available on Bioproject (accession number PRJNA222758)

### Differential gene expression analysis

In order to identify coral transcripts that differed significantly in their expression between the two disease resistance phenotypes (resistant vs. susceptible), differential expression analysis was performed using the R package DESeq2 [21]. We performed a two-factor negative binomial, Wald test to compare RNA-seq gene expression patterns between experimental conditions using a two factor design with an interaction (design = ~ resistance + exposure + resistance*exposure). Resistance (resistant vs. susceptible genotypes) and exposure (disease graft, healthy graft and no-graft control) as the main factors with resistance*exposure as the interaction term. This two-factor model allowed us to identify transcripts significantly associated with resistance, exposure, both factors (resistance + exposure), as well as significant interactions (resistance*exposure). Size factor normalization, variance reduction and outlier detection were performed on the expression data in DESeq2. For each transcript, the fold change is reported as the average normalized read counts per treatment group relative to a baseline (susceptible for the resistance treatment, no-graft control for the exposure treatment). Genes were considered to be significantly differentially expressed at a false discovery rate (FDR) adjusted p-value (adj-p) < 0.05 [22].

To visualize the patterns of differential expression between resistant and susceptible coral genotypes, a clustered heat map of the differentially (DE) transcripts was created using log-transformed normalized count data in GENE-E [23]. Hierarchical clustering was performed using Pearson's correlation metric.

## Results and Discussion

**Fig 2:**
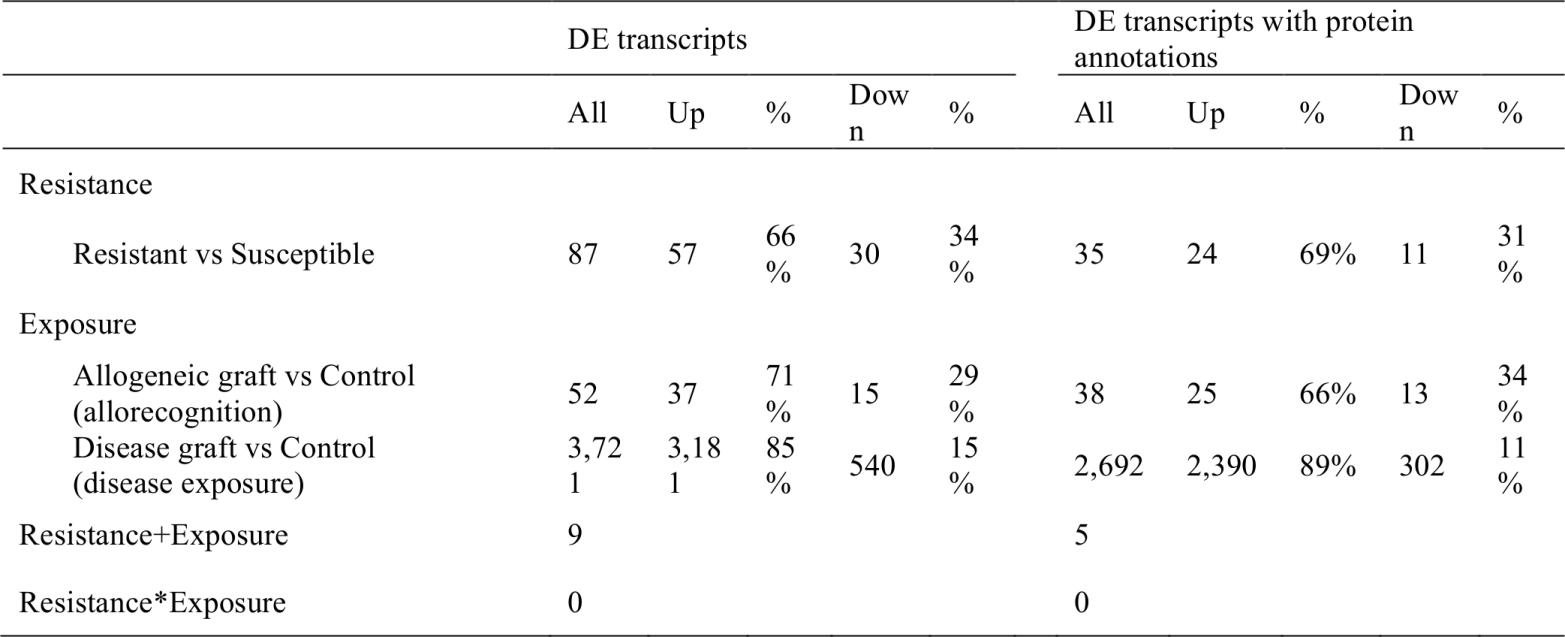
Differential expression analyses of RNA-seq data for staghorn coral resistance phenotypes and exposure treatments. Number of coral transcripts exhibiting significant differential expression (adj p-val<0.05) due to the resistance phenotype (resistant vs susceptible) and to exposure (allogeneic graft vs control; disease graft vs control). Only five transcripts were significant in both treatments (R+E, see also S1 Table). No significant interaction was found between resistance and exposure (R*E = 0).

The average number of uniquely mapped reads per sample was 3,238,039 (±1,037,905) across all 17 samples, 3,852,932 (±525,993) for resistant corals and 2,359,619 (± 962,951) for susceptible corals. Of these, the average number of mapped coral reads per sample was 1,443,485 (±614,646) for resistant samples and 2,305,935 (±301,156) for susceptible samples. Two-factor GLM analyses of RNA-seq gene expression patterns comparing staghorn coral resistant phenotypes (WBD resistant vs. susceptible corals) and exposure treatments (disease graft vs. allogenic graft vs. control) identified 87 (57 up- and 30 down-regulated) coral gene transcripts that were significantly differentially expressed (DE) due to the resistance phenotype, 3,721 (3,181 up- and 540 down-regulated) DE transcripts due to disease exposure, and 52 (37 up- and 15 down-regulated) DE transcripts due to allogeneic grafting (Fig 2). Out of the 87 DE disease resistance transcripts, 78 transcripts differed only due to resistance while the remaining nine transcripts (N = 5 annotated) differed due to disease exposure as well (resistance + exposure). These five transcripts included two 40S ribosomal protein (S9 and S12) and three predicted proteins from the sea anemone *Nematostella vectensis*: v1g198603- a putative membrane receptor- v1g214092-a putative hydrolase- and v1g206329- a transcript containing epidermal growth factor (EGF)-like, WSC, Laminin G-3, Pentaxin and Polycystin-1, Lipoxygenase, Alpha-Toxin (PLAT) domains. All these transcripts exhibited opposite expression changes in the two treatments: v1g206329 was up-regulated in resistant corals but down-regulated in WBD-exposed corals, v1g214092, v1g198603 and the two ribosomal proteins were down-regulated in resistant corals but up-regulated in WBD-exposed corals (see S1 Table).

No transcripts showed significant interactions between the two factors (resistance x exposure). This indicates that 90% (78 out of 87) of DE transcripts associated with disease resistance behave independently of disease exposure (i.e. whether they were grafted with diseased or healthy coral or received no graft at all) and thus represent constitutively expressed differences between resistant and susceptible staghorn coral genotypes.

**Fig 3:**
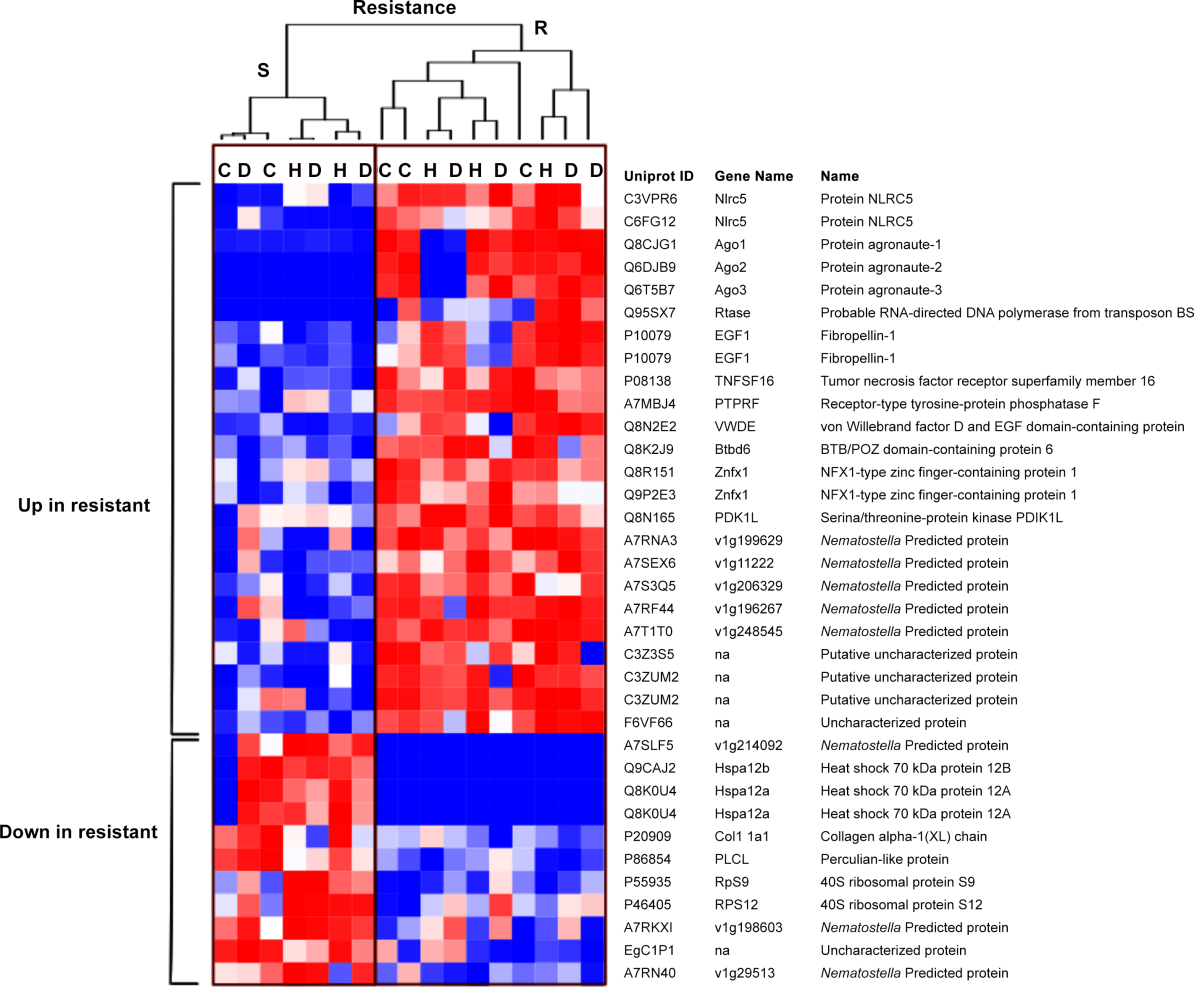
Gene expression analysis of resistant (R) and susceptible (S) corals exposed to allogeneic tissue and active White Band Disease (WBD). Clustered heatmap for gene expression of the 35 annotated differentially expressed genes that differ between resistant (R) and susceptible (S) corals exposed to WBD grafts (D), healthy grafts (H) and unexposed (no graft) controls (C). Hierarchical clustering was performed using Pearson correlation metrics and indicates similarity between the different samples (columns), with **resistant and susceptible samples forming different clusters.** Red corresponds to up-regulated genes, blue indicates down-regulation. The expression level for each transcript was calculated as log2 fold change of normalized count data. Asterisks indicate transcripts that were DE in both factors (resistance + exposure). See also S1 Table.

Out of the DE expressed genes with strong protein annotations (tBlastx e-value < 10^−5^), 35 DE transcripts were associated with disease resistance (24 up- and 11 down-regulated), 2,692 were associated with disease exposure (2,390 up- and 302 down-regulated) and 38 were associated with allogeneic grafting (25 up- and 13 down-regulated). In order to characterize the genetics of disease resistance, we focused on the 35 annotated coral transcripts that were DE between resistant and susceptible genotypes. The overall expression profiles of resistant and susceptible corals are displayed in Fig 3, representing the clustered heat map of normalized log-transformed count data for these 35 DE transcripts. Interestingly, clustering and functional annotation indicated that none of the 35 DE transcripts associated with disease resistance were previously identified as key mediators of staghorn coral immune response against WBD [16]. This, together with the absence of DE transcripts with significant interactions between resistance and exposure indicate that the genetic basis of WBD resistance differs from the immune response elicited by disease (pathogen) exposure. This may also explain why the majority of transcripts associated with resistance were not influenced by exposure [i.e. diseased or healthy (allogeneic) grafts].

Resistant corals differed from susceptible corals in their higher expression of gene transcripts associated with RNA interference-mediated gene silencing (RNAi) and antiviral defence, including three Argonaute proteins, AGO1 (3.6 fold up), AGO2 (3.5 fold up) and AGO3 (3.6 fold up), and two NOD-like receptor family CARD domain containing 5 (NLRC5) (3.0–2.1 fold up).

Elevated Argonaute expression in resistant corals suggests there is link between RNAi-mediated gene silencing and disease resistance in staghorn corals, but it is not yet clear if the relationship is direct, as an antiviral defence via small interfering RNA (siRNA), or indirect, as part of host gene regulation via microRNA (miRNA). Corals and their cnidarian relatives possess the key components of the RNAi machinery [24] and a diverse repertoire of miRNAs [25]. Argonaute proteins have been linked to pathogen resistance and immunity in animals and plants. For example, in *Drosophila*, AGO2 mutants are more susceptible to viral infection [26]. In plants, AGO2 expression is elicited by bacterial exposure and promotes the production of antimicrobials via miRNA-dependent gene regulation [27]. Data directly linking Argonaute expression to disease resistance is limited. Yet, in *Arabidopsis*, AGO4 regulates resistance to pathogens via RNA-directed DNA methylation [28], an epigenetic mechanism of transcriptional gene regulation.

NLRC5 is a Pathogen Recognition Receptor (PRR) in the NOD-like receptor (NLR) family that detects intracellular pathogens and regulates inflammatory cytokines production [29]. In vertebrates, NLRC5 can mediate antiviral defences [30], regulate inflammation and immunity via NF-kB signalling in the cytoplasm [31], and even act as transcriptional regulator of major histocompatibility complex (MHC) class II genes [32]. While NLRs are broadly considered to be important innate immune regulators [33], relatively little is known about their roles in invertebrates, in part because they are absent in model organisms like *Drosophila* and *Caenorhabditis elegans* [34]. In plants, nucleotide-binding site–leucine-rich repeat (NBS–LRR) proteins, which are structurally similar to NLRs, detect pathogens and can confer disease resistance [35]. Interestingly, two genetic examples of host disease resistance (R) genes in plants involve endogenous siRNA-mediated gene silencing and miRNA-mediated regulation of auxin signalling [36]. Corals and other cnidarians possess an unusually high diversity of NLRs compared to other invertebrates [37], but no direct evidence for their immune function has been reported until now. Up-regulation of NLRs in resistant staghorn corals suggests they play an important role in pathogen detection and immunity, possibly even allowing resistant corals to avoid pathogen infection.

One surprising feature of the gene expression patterns in the resistant staghorn corals was the down-regulation of 70 kDa heat shock proteins, specifically heat shock 70 kDa protein 12B (HSPA12b) (6.9 fold down) and heat shock 70 kDa protein 12A (HSPA12a) (N = 2; 8–5.8 fold down) (Fig 2). Heat shock proteins (HSPs) are ubiquitous molecular chaperones that regulate inflammation and immunity [38]. Members of this family, in particular Hsp70, are also induced during thermal stress to protect proteins and cell membranes from the damage caused by incorrect protein folding and aggregation [39]. In corals, increased HSPs expression has been documented in response to thermal stress during coral bleaching and due to increasing acidification [40], but not yet due to pathogen exposure [16]. While it is not clear why HSP70 would be down-regulated in disease resistant corals, recent transcriptomic data from thermally resilient *A*. *hyacinthus* corals living in high temperature environments have been shown to exhibit less change in HSPs expression in response to heat stress compared to heat sensitive colonies [6]. This reduced heat stress reaction may be a result of lower levels of intracellular stress in heat-tolerant colonies, suggesting that that the down-regulation of HSPs in disease resistant staghorn corals may indicate increased thermal resilience as well.

## Conclusions

Transcriptome-wide analysis of the gene expression patterns underlying disease resistance in the staghorn coral *A*. *cervicornis* demonstrates that resistance to WBD infection is conferred by the constitutive expression of multiple gene pathways, including up-regulation of RNAi-mediated gene silencing and down-regulation of HSPs. How the up-regulation of Argonaute and NLRC5 confers disease resistance in staghorn corals is unknown. Recent research indicates that WBD infection can be blocked by antibiotic treatment, indicating that the pathogen is bacterial [9, 10]. It is possible that WBD infection involves both bacterial and viral pathogens [9], and that the up-regulation of Argonaute in resistant corals is being used to respond to exogenous double-stranded RNA (dsRNA) via siRNA. However, NLRC5 and Argonaute were constitutively up-regulated and not up-regulated due to pathogen exposure. Thus, a second and more intriguing possibility is that the up-regulation of Argonaute transcripts in disease resistant corals is used for miRNA-based post-transcriptional regulation. This suggests a novel role for miRNA-directed gene silencing in cnidarian immunity, which would be similar to the links between miRNA and pathogen resistance in plant immunity. Down-regulation of HSPs in disease resistant staghorn corals indicates a possible link between lower HSP expression and coral disease resistance. This finding, coupled with recent evidence for constitutive HSPs expression in thermally resilient pacific *Acropora* corals [6], suggests the intriguing possibility that disease resistant corals may show increased thermal resilience as well. The ability to identify resistant staghorn corals in remnant populations across the Caribbean using these strong, constitutive differences in gene expression at Argonaute and HSP transcripts could also serve as a valuable conservation tool and aid on-going nursery and out-planting efforts that are currently underway for this endangered species.

## Supporting Information

S1 Table Expression values of significantly differentially expressed transcripts in resistant corals. (XLSX)
